# Septins: molecular partitioning and the generation of cellular asymmetry

**DOI:** 10.1186/1747-1028-4-18

**Published:** 2009-08-26

**Authors:** Michael A McMurray, Jeremy Thorner

**Affiliations:** 1 Division of Biochemistry and Molecular Biology, Department of Molecular and Cell Biology, Room 16, Barker Hall, University of California at Berkeley, Berkeley, CA 94720-3202 USA

## Abstract

During division, certain cellular contents can be distributed unequally; daughter cells with different fates have different needs. Septins are proteins that participate in the establishment and maintenance of asymmetry during cell morphogenesis, thereby contributing to the unequal partitioning of cellular contents during division. The septins themselves provide a paradigm for studying how elaborate multi-component structures are assembled, dynamically modified, and segregated through each cell division cycle and during development. Here we review our current understanding of the supramolecular organization of septins, the function of septins in cellular compartmentalization, and the mechanisms that control assembly, dynamics, and inheritance of higher-order septin structures, with particular emphasis on recent findings made in budding yeast (*Saccharomyces cerevisiae*).

## Review

### Overview: Jumping Through Hoops

After anaphase, cytokinesis completes the process of producing two cells from one. For proliferation to occur, each daughter cell must receive at every such mitosis all of the requisite components essential for subsequent division. During development, by contrast, certain daughter cells inherit particular cellular constituents differentially, which can influence their fate. Within non-dividing cells, establishment of cellular asymmetry ("polarity") requires spatial segregation of molecular components, and this selective partitioning may be a fundamental feature of life [1]. Despite its universal importance, many aspects of how such subcellular asymmetry is generated remain poorly understood at the mechanistic level. In a number of biological contexts, a set of conserved proteins, called septins, has emerged as a central player in polarity determination and asymmetric cell division.

The septins are a family of GTP-binding proteins found in nearly all eukaryotes (higher plants are the main exception) [2]. A given septin assembles with other septins into a linear hetero-oligomeric complex ("rod"), and rods can associate end-to-end to form longer polymers ("filaments") [3]. For example, the *S. cerevisiae *rod capable of polymerization *in vitro *is a hetero-octamer composed of four different gene products in the following order: Cdc11–Cdc12–Cdc3–Cdc10–Cdc10–Cdc3–Cdc12–Cdc11 [4]. Targeted localization directs assembly of septin ensembles at particular sites, and septin-containing structures have been implicated in a wide variety of cellular processes [5]. Septin-based structures seem to perform, in essence, two non-catalytic roles. First, septin structures serve as scaffolds for the recruitment of non-septin factors, *i.e*., they participate in cell morphogenesis and cell division via their direct physical interaction with various enzymes and regulatory proteins. Second, septin structures that are closely associated with membranes can serve as barriers that restrict the movement of certain integral membrane proteins, *i.e*., localization of such membrane proteins is septin-dependent, but does not seem to involve their stable binding to the septins [6-8].

**Figure 1:**
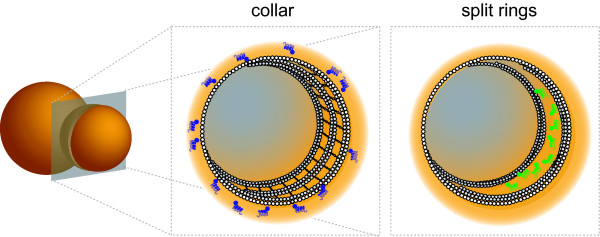
**Models for septin organization and diffusion barrier function in the collar and split rings assemblies at the yeast bud neck**. This model, based on experimental observations and considerable speculation, illustrates views of the mother-bud neck from a position within the bud (approximated by the *gray plane*), showing the plasma membrane (*orange*), globular septin G domains (*white balls*), and non-septin proteins (*blue, green*) integral to the plasma membrane and restricted to discrete cortical domains via septin-based diffusion barriers (*e.g*., Sec3 [6,7]). Prior to cytokinesis, the septins at the bud neck comprise a filamentous collar (*left view*), retaining Sec3 in the bud (*blue*). The beginning of cytokinesis is marked by splitting of the collar into two discrete rings (*right view*), followed by septin-dependent accumulation of Sec3 (*green*) and other cytokinesis factors within a cortical neck compartment, concomitant with actomyosin ring contraction and growth of the chitinous septum. During this transition, the C-terminal extensions (*wavy lines*) projecting orthogonally from the filaments in the collar rotate 90°, allowing for greater side-by-side compaction of the filaments.

Both functions are found in cells of the budding yeast *Saccharomyces cerevisiae*, wherein a collar of septin filaments assembles at the isthmus between a mother cell and its daughter (the bud) (Figure 1). On the one hand, this collar has a scaffold role. For example, two different protein kinases, Cla4 and Hsl1, possess septin-binding domains that mediate their high-affinity association with septin filaments, both *in vivo *and *in vitro *[9,10]. On the other hand, the filamentous septin collar at the bud neck also has a barrier role because it prevents the free diffusion of specific plasma membrane (PM) [6-8], endoplasmic reticulum (ER) [11], and nuclear envelope (NE) [12] proteins between the mother cell and its daughter. Thus, by imposing (via either mechanism) a highly anisotropic distribution of multiple cortical factors, the septin collar is critical for the coupling of normal cell morphogenesis with the execution of the cell division cycle and, without it, cell growth is no longer restricted to the bud [6]. Intriguingly, in a variety of specialized non-dividing cells, septins accumulate in highly polarized regions at sites that are appropriately situated to recruit specific factors to the cortex and/or to restrict the diffusion of other factors already at the cortex. Examples include: on the flanks of the projection formed by a haploid yeast cell in response to its exposure to a peptide mating pheromone [13,14]; at the base of the spines that project from the dendrites in neurons [15,16]; and within the sperm annulus, a structure that separates the head and midpiece of a mature spermatozoan from its tail [17,18].

Mechanistically, how septins create a barrier to diffusion along a membrane remains largely unknown. However, the ability of septin complexes to polymerize into filaments provides one reasonable possibility. For example, at the EM level, the septin collar at the bud neck appears to comprise a highly ordered array of continuous circumferential filaments ("hoops") [19] and these are present at the stage of the yeast cell cycle when diffusion of cortical components between a mother and its bud is demonstrably restricted [6]. Yeast septin rods (either isolated from *S. cerevisiae *[20] or prepared by expression in and purification from *E. coli *[4,21-23]) are able to self-assemble under the right conditions (salt concentration ≤ 150 mM) into filaments that strikingly resemble the neck filaments, suggesting that these septin filaments are themselves the primary constituents of the collar hoops. Indeed, indirect immunofluorescence using anti-septin antibodies [24] and examination of cells expression GFP-tagged septins [25] confirm that the collar contains the vast majority of the septins present in a budded cell. Moreover, the collar filaments are closely apposed to the PM [19]. It has been suggested that membrane association of septins is mediated via their interaction with phosphatidylinositol-4,5-*bis*phosphate (PtdIns4,5P_2_) in both mammalian cells [26] and yeast [27]. Consistent with this property of the septins and their location in a budded cell, there is evidence that PtdIns4,5P_2 _is enriched in the PM at the bud neck [28]. Furthermore, forced wholesale conversion of the PM PtdIns4,5P_2 _pool to PtdIns3,4,5P_3 _causes the detachment of septins from the bud neck and the formation of coils and rings in the cytosol [29]. Preparations of human septins are purportedly capable of remodeling large synthetic membrane vesicles into tubular projections by wrapping around the tubes in a PtdIns4,5P_2_-dependent manner [30]; however, the possibility of contamination by other proteins (like ESCRT III components) capable of membrane tubulation has not been scrupulously eliminated. In any event, the clear-cut implication of these collective findings is that, by associating tightly with the PM, continuous septin filaments in the collar at the bud neck could act like the fences in a corral to physically constrain the movement of both lipids and proteins, thereby preventing their free passage between a mother cell and its bud [31,32] (Figure 1). The septin filaments in the collar appear to coat the PM at the neck, but project less than 10–20 nm into the cytosol; at least one septin-associated peripheral membrane protein, Bud6, is required to impose the ER and NE barriers, but not the PM barrier [11,12], indicating that different factors are involved in establishing the septin-dependent diffusion barriers at the PM and at other membranes.

**Figure 2:**
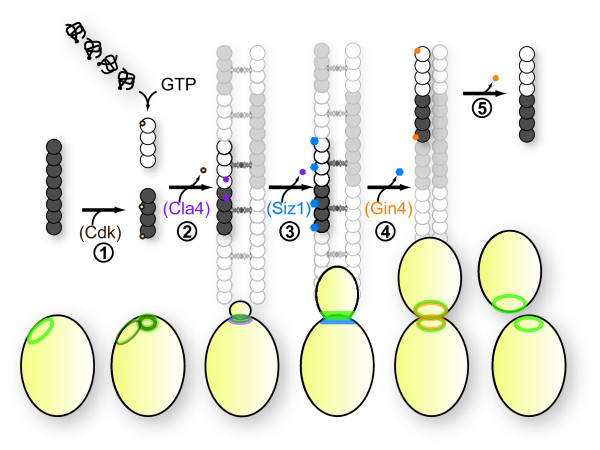
**Model for major transitions in septin assembly and modification state during the yeast budding cycle**. Subcellular septin localization (*green*) during the cycle cycle is accompanied by changes in the organization and covalent modification of septin subunits (*grey and white balls*). (1) In the G1 phase, hetero-octamers of septin subunits (*gray balls*) within the "old" ring persisting from the previous cell division are subject to phosphorylation (*brown dots*) by G1 cyclin-activated cyclin-dependent kinases (Cdks). This modification on certain subunits (*e.g*., Cdc3 [50]) promotes dissolution of the old ring, permitting relocalization to a new ring at the next budding site. Newly translated septin polypeptides fold, bind GTP, and assemble into sub-octameric complexes (both Cdc11—Cdc12—Cdc3—Cdc10 and Shs1—Cdc12—Cdc3—Cdc10 tetramers, in this model; *white balls*) that remain stably associated throughout the lifetime of the proteins. Co-incorporation of pre-existing and newly-synthesized subcomplexes precedes (2) phosphorylation by Cla4 (*purple dots*) of certain subunits (*e.g*., Cdc10 [10]), which promotes assembly into an organized array of filaments at the neck of the emerging bud. (3) Prior to cytokinesis, SUMO (*blue hexagons*) is attached to certain subunits in a Siz1- and Siz2-dependent manner only on the mother side of the neck [63,76,77]. (4) During mitosis, septin phosphorylation (*orange dots*) by mother-side Gin4 (and presumably by its sister protein kinase Kcc4 on the bud side) promotes splitting of the septin collar. (5) Following the completion of cytokinesis and cell separation, septin filaments disassemble into hetero-octamers; residual ring-like septin deposition may reflect persistent self-reinforcing organization of PtdIns4,5P_2 _and septin-binding transmembrane proteins at the cell cortex. Note that removal of each septin modification upon completion of the preceding transition is speculative, but consistent with the role ascribed, for example, to the action of the Rts1-containing isoform of PP2A [53], and with the ability of old and new subunits to populate all septin-containing structures in a given cell [42].

Initially, several observations were difficult to reconcile with the corral model for how septins exert a barrier function. However, these objections turn out to be superficial in light of more recent information. For example, one concern raised arises from the fact that the septin-dependent diffusion barrier that restricts diffusion of factors at the bud neck is maintained during cytokinesis, even though at this stage of the budding yeast division cycle the prominent array of neck filaments at the isthmus become virtually undetectable, at least by EM [19]. However, when visualized by indirect immunofluorescence or using fluorescently-tagged septins, thin septin-containing "rings" are observed on both the mother and bud sides of the isthmus [33] (Figure 2). Thus, if these septin-based rings also comprise continuous filamentous hoops (Figure 1), they could clearly suffice to provide barrier function, even though they are difficult to observe via EM. Indeed, formation of these rings performs a function that is essential for cytokinesis because if these rings are allowed to form, but then artificially disrupted (by use of a heat-sensitive mutation that causes septin filament disassembly at the restrictive temperature), cytokinesis fails [7].

A second concern raised about the corral model has to do with a dispute about the orientation of the bud neck filaments relative to the mother-bud axis. For a corral to prevent movement of factors from one side of the isthmus to the other, at least some of the filaments in the collar should run perpendicular to that axis, *i.e*., circumferential to the neck (Figure 1), consistent with the interpretation of the original EM images in which the filaments were first detected [19]. However, it has been posited, instead, that the filaments run parallel to the mother-bud axis, and that the filaments only appear circumferential because, according to this view, the filaments are in perfect side-by-side register and their constituent septin subunits have differential avidity for the stain used for EM visualization [34,35]. To attempt to bolster this argument, the orientation *in vivo *of septin filaments containing GFP-tagged Cdc12 or Cdc3 was examined by measurement of the fluorescence polarization of this fluorophore, compared to a "standard" (bundled filaments of the same proteins prepared by purification and assembly *in vitro*) [36]. By this criterion, the filaments in the collar appeared to be oriented parallel to the mother-bud axis. Interestingly, in these studies, as judged by a 90° shift in the fluorescence anisotropy of the GFP-tagged septins *in vivo *at the onset of cytokinesis, the orientation of the septin filaments seemed to undergo a 90° rotation when the collar is split into two rings [36], consistent with the rings now comprising circumferential filaments (as would be needed for a septin-based corral). Yet, the barrier function of the septin collar is also exerted prior to cytokinesis; so, the question remained of how filaments parallel to the mother-bud axis can do so. In the fluorescence polarization studies, it was assumed that the orientation of the GFP relative to the septin to which it is attached remains fixed and that the chimera behaves as one rigid object. However, more recent ultrastructural analysis has demonstrated that the carboxyl-terminal portion of the septin proteins (to which the GFP was attached in the polarized fluorescence experiments) is flexible and able to rotate freely relative to the filament axis [4,22,37]. Thus, the most parsimonious conclusion that reconciles the available EM and fluorescence polarization data is to view the septin collar as an array of circumferential filaments, as originally proposed, which is resolved at the onset of cytokinesis into a pair of rings that are also composed of circumferential filaments, but in which the flexible septin carboxy termini have undergone a 90° rotation (Figures 1 and 2). Accordingly, at both stages of the cell cycle, the collar and the rings have the same underlying mechanistic basis for exerting a diffusion barrier function. The shift in orientation of the septin tails may simply reflect a conformational change induced by their association with different sets of proteins and/or lipids, or different types or extents of cell cycle stage-specific post-translational modifications.

A third objection to the idea that an essential function of the septins is the formation of circumferential filaments that establish a diffusion barrier was a study that concluded that assembly of septin filaments *per se *was not essential for *S. cerevisiae *cell division [20]. The data in this study that seemed the most persuasive at the time was the proliferative ability of cells that lack a particular septin (either Cdc10 or Cdc11) and in which no prominent array of neck filaments was visible by EM, and from which purified septin complexes were unable to polymerize into filaments *in vitro *[20,23,27,38-40]. Considering that we now appreciate that, in yeast, the building block of filaments is a linear (single subunit-wide) hetero-octameric rod that polymerizes end-on-end [4,22], deletion of any single subunit might be expected to preclude polymerization. However, when expressed in and purified from bacteria, complexes of yeast septins lacking either Cdc10 or Cdc11 have been reported to form filaments *in vitro*, although the protein concentrations required are higher and the resulting filaments are less organized than observed for the complete four-subunit complex [21,23]. Moreover, although the majority of *cdc10*Δ cells in the study of Frazier *et al*. did not exhibit a pronounced array of neck filaments visible by EM, one cell did display a repeated pattern of cortical profiles reminiscent of neck filaments [20]. Furthermore, in most *cdc10*Δ cells, the remaining septins form an apparently continuous collar at the bud neck [20,23]. Hence, there is evidence to suggest that septin filament assembly *in vivo *is more forgiving to the absence of constituent subunits than previously thought and, in such cases, detection of neck filaments may require less harsh fixation methods and/or more sensitive techniques. Thus, the findings initially reported by Frazier *et al*. [20] do not compellingly exclude the possibility that septin filaments form and are essential for proliferation, and that an essential function of these filaments is the creation of a diffusion barrier.

A fourth finding that has been raised as an argument against the necessity for formation of circumferential neck filaments was the fact that in cells lacking a particular bud neck-associated protein kinase, Gin4, the septins appear at the neck as a series of roughly evenly spaced bars running along the mother-bud axis (a "collar of bars") [35], instead of the uniform, hourglass-shaped distribution of septins seen in wild-type cells. This arrangement clearly seems incompatible with a corral-type mother-bud diffusion barrier, yet cells containing such abnormal septin-based structures are able to proliferate. The "collar of bars" phenotype is only displayed by a small fraction of the population of *gin4*Δ cells, but is more penetrant when *gin4*Δ cells are grown at high temperatures. At any given time, more cells display this aberrant septin assembly than display cytokinesis defects, which was taken as evidence that such a collar of septin bars was sufficient for septin function in cell division [35]. On this basis, it was concluded that a hoop-like arrangement of the filaments in the collar is not essential for cell division in budding yeast. The major flaws in this logic are the assumptions that, for every cell found to have a collar of bars, this aberrant structure is the best that the cell will ever assemble during that particular attempt at cell division, and that such cells actually then divide. In fact, however, time-lapse microscopy of *gin4*Δ cells expressing a GFP-tagged septin reveals that, in the cells where such bars form, division is delayed, followed either by resolution into a uniform collar and resumption of cytokinesis, or a terminal arrest if the aberrant bar structures persist (our unpublished observations). Thus, it appears that only a uniform septin assembly consistent with circumferential neck filaments is capable of supporting proper cell division.

### Versatile Frameworks for Cellular Compartmentation

Cellular subdivision is not an event restricted to the act of cytokinesis during mitotic proliferation. In yeast, for example, meiotic nuclear division is accompanied by a cellularization process, dubbed sporulation [41]. Each one of the four haploid nuclei resulting from meiosis becomes surrounded by a new plasma membrane and cell wall, and these envelopes also encase other cellular components necessary for spore viability and germination. During yeast sporulation, septins are found in a series of structures that assemble at the leading edge of the developing spore membrane [39,42,43], where they appear strategically positioned to direct proper localization of the enzymes and regulatory factors directly responsible for spore membrane and wall deposition. PtdIns4,5P_2 _is highly enriched in these pre-spore membranes [44], suggesting some common features of the mechanism by which septins interact with those membranes that undergo remodeling during mitotic and meiotic division. However, how this localized recruitment is achieved and why the septins concentrate only in membrane regions undergoing active reorganization is not well understood. Septins appear to interact intimately with microtubules in sporulating cells [43] and phosphoprotein phosphatase 1 (PP1)-dependent dephosphorylation of as yet unidentified substrates is also critical for proper septin organization during sporulation [41,45,46]. Indeed, septin dynamics during spore formation cannot all be explained by the distribution of PtdIns4,5P_2 _because this lipid is also present in other parts of the developing spore membrane during sporulation [44] and, likewise, is present in other regions of the plasma membrane during mitotic division. It is possible that the converse model may apply. If septins restrict the diffusion of membrane phospholipids, then the observed PtdIns4,5P_2 _enrichment at the prospore membrane may be imposed by the concentration of septins there, and not *vice versa*. However, it is not known if septin-based structures impose a diffusion barrier at the prospore membrane, or whether the sporulation-specific septin complexes, which contain two additional meiosis-specific septins, Spr3 and Spr28 [39,47], polymerize into filaments.

### Partitioning of a Complex Protein Assembly during Cell Division: Septins as a Paradigm

As described above, septin-based structures play important roles in cytokinesis, cell compartmentalization, and cell polarity. At the same time, these elaborate multi-protein ensembles provide an opportunity to understand how complex supramolecular structures are segregated during cell division [33]. During each division of a yeast cell, the five mitotically-expressed septins (Cdc3, Cdc10, Cdc11, Cdc12 and Shs1) co-assemble into a ring that marks the bud site. Concurrently with bud growth, the ring expands into the hourglass-shaped collar that lines the isthmus between the mother cell and its daughter. At cytokinesis, the collar transforms into two rings that demarcate each side of the bud neck (Figures 1 and 2). As mentioned in the preceding section, two additional septin genes are turned on during sporulation, and their products (Spr3 and Spr28) co-assemble with some of the mitotically-expressed subunits (and exclude others) [42], forming a series of structures that ultimately disappear when, upon germination, a spore resumes mitotic division [33]. Below, we consider what is currently known about the mechanisms by which the septin proteins and the structures of which they are composed are inherited through mitotic and meiotic cell divisions.

### Septin Modifications Accompany and Direct Higher-Order Organizational Transitions

Certain cellular factors cannot persist and be passively segregated into daughter cells because their presence would be incompatible with orderly cell division or with the onset of a developmental transition. Cyclins are a good example. These proteins drive the events of mitosis and cytokinesis by directing cyclin-dependent kinases (Cdks) to the substrates whose phosphorylation is rate-limiting for these events. Hence, execution of the cell cycle requires that cyclins be destroyed in the proper temporal and spatial order, thereby yielding new-born daughters competent to undergo terminal differentiation or to initiate their own first division (by commencing reiteration of the same program of cyclin expression).

All five septins (Cdc3, Cdc10, Cdc11, Cdc12 and Shs1) in budding yeast persist throughout the cell division cycle and co-localize indistinguishably at every cell cycle stage [33]. Therefore, unlike cyclins, the complement of septins does not undergo any dramatic change during passage through each cell cycle transition. However, the septins do undergo multiple cell cycle stage-specific modifications that coincide with the dramatic reorganizations of septin-based structures that occur concurrently with progression through the cell division cycle (Figure 2). Thus, it seems reasonable to propose that these modifications affect intermolecular interactions among the septins themselves and/or association of septins with other cellular factors, thereby systematically altering the architecture and components present in septin-based structures at different stages of the cell cycle.

Concomitantly with exit from G1, Shs1 is phosphorylated on a number of Ser and Thr residues by two different Cdks (Cdc28 and Pho85), which may drive septin complexes to assemble into the ring that marks the site of bud emergence [48,49] and/or install other marks on Shs1 important for subsequent modifications. Later in the cell cycle, Cdc10 is phosphorylated on Ser256 by a bud neck-associated protein kinase, Cla4 (Table 1), that contains a PH domain able to bind PtdIns4,5P_2 _[28], which appears to promote efficient septin collar formation at the bud neck [10]. Coincident with the onset of cytokinesis and splitting of the collar into two rings, Shs1 is phosphorylated on a combination of residues different from those modified in G1, probably by another bud neck-associated septin-binding protein kinase, Gin4 [49]. Immediately after cell separation, Cdc3 is phosphorylated in a Cdk -dependent manner, which seems to promote disassembly of each "old" ring (one inherited by the mother and one by the new-born daughter) because mutations that prevent this modification delay old ring disassembly [50]. Interestingly, Cdk-mediated phosphorylation of specific residues on the Cdc11 ortholog in *Candida albicans *coincides with and is functionally involved in hyphal development [51], an alternative mode of growth required for pathogenesis by this otherwise yeast-form fungus.

**Table 1:** Protein-modifying enzymes and their targets at the *Saccharomyces cerevisiae *bud neck

**Gene product**	**Closest mammalian ortholog**	**Function**	**Neck substrate(s)**	**Reference(s)**
*Protein kinases*				
Cbk1	NDR2	NDR family; cell wall morphogenesis, cell separation	Ace2	[79]
Cdc5	PLK2	Polo/PLK family; cell cycle regulation, cytokinesis, mitotic exit	Swe1	[80]
Cdc15	MST1	STE20-like family; activated by GTP-bound Tem1, late nuclear division, mitotic exit	Dbf2	[81]
Cdc28	CDK1	Catalytic subunit of Cdk1; cell cycle progression	Cdc3 Shs1 Swe1	[48,50,82]
Clb2	Cyclin B2	B-type cyclin; regulatory subunit for Cdc28; cell cycle progression	---	[83]
Dbf2 Dbf20	LATS1	NDR family; anaphase-telophase, mitotic exit	Cdc14	[84]
Elm1	LKB1	Upstream activator of Snf1 and other AMPK-like protein kinases involved in septin assembly	Gin4 Hsl1 Kcc4	[85,86]
Gin4	BRSK1	Nim1-related and AMPK-like family; septin collar assembly	Shs1 Swe1?	[49]
Hrr25	CKIδ	Casein kinase I family; located throughout the cell, but concentrated at bud tip and bud neck	?	[87]
Hsl1	BRSK2	Nim1-related and AMPK-like family; morphogenesis checkpoint, Swe1 degradation	Shs1 Swe1	Trott AE, Gullbrand B & Thorner J, unpublished results
Kcc4	BRSK1	Nim1-related and AMPK-like family; septin collar assembly	Swe1?	[88]
Kin4	MARK4	AMPK-like family; negatively regulates mitotic exit in response to a misaligned spindle	Gic2? Hsl1?	[89,90]
Mob1	MATS1	Regulatory and substrate targeting subunit of Dbf2 (and Dbf20)	---	[91]
Mob2	MOB1A	Regulatory and substrate targeting subunit of Cbk1	---	[91]
Pcl1 Pcl2 Pcl9	Cyclin A/B	AB-type cyclins; regulatory subunits for Pho85; cell morphogenesis	---	[92-94]
Pho85	CDK5	Catalytic subunit; transcription, cell morphogenesis	Bni4 Cdc3 Cdc10 Cdc12	[33,95]
Pkc1	PRK2	Cell wall integrity; activated by GTP-bound Rho1, localizes to bud neck late in the cell cycle before cell separation	?	[96]
Rad53	CHK2	DNA damage checkpoint	Shs1 Cdc12	[97]
Swe1	WEE1	Morphogenesis checkpoint	Cdc28	[98]
Yck2	CKIγ	Casein kinase I family; a peripheral plasma membrane protein anchored by C-terminal palmitoylation	?	[99]
*Phosphatases*				
Afr1	PHACTR4	Regulatory and substrate targeting subunit of phosphoprotein phosphatase (PP) 1; septin reorganization during mating projection formation	---	[100]
Cdc55	B55β2	B-type regulatory and substrate targeting subunit of PP2A	---	[101]
Glc7	PP1	Catalytic subunit of PP1	?	[102]
Pph21 Pph22	PP2ACα PP2ACβ	Catalytic subunit isoforms of PP2A	?	[101,103]
Rts1	B56δ1	B'-type regulatory and substrate targeting subunit of PP2A	Shs1	[53]
Tpd3	PR65α	A-type regulatory (scaffold) subunit of PP2A	---	[103]
*Other modifiers*				
Grr1	FBXL20	F-box protein for substrate recognition by an SCF (Skp1—Cdc53—Cdc34)-type ubiquitin-protein ligase (E3), targets landmark proteins for destruction after septin ring site is established	Gic1? Gic2	[104,105]
Hsl7	JBP1	PRMT5; type II protein-arginine methyltransferase that generates symmetric N, N'-dimethylarginine residues in its substrates	Swe1?	[106,107]
Siz1 Siz2/Nfi1	PIAS2 PIAS4	RING-like domain-containing SUMO-protein ligases (E3); responsible for mother side-specific attachment of SUMO to septins in the collar at the neck at the onset of anaphase	Cdc3 Cdc11 Shs1	[61,76]
Mms21	-none-	U box domain-containing SUMO-protein ligase (E3); capable of attaching SUMO to septins when Siz1 & Siz2 absent	Cdc3 Cdc11 Shs1	[63]
Smt3	SUMO-1	Small ubiquitin-like modifier; may contribute to regulating disassembly of septin collar and/or association of other proteins with septins in the collar	---	[108]

One way to remove structure- and stage-specific subunit modifications would be to actively reverse them via the action of enzymes that catalyze removal of the modifications. Alternatively, like cyclins, modified septins could simply be destroyed and resynthesized *de novo *at the appropriate time. Current evidence demonstrates that, in the case of septins, an orderly program of reversible modification (rather than periodic synthesis and degradation) drives the observed changes in organizational state. In mitotically dividing yeast cells, septin polypeptides exhibit a very long half-life [42,52] and are re-incorporated into every septin-containing structure through multiple successive cell divisions [42]. Furthermore, as mitosis ends, a targeting subunit for phosphoprotein phosphatase 2A (PP2A), Rts1, localizes this enzyme to the split septin rings, promoting dephosphorylation of Shs1 [53]. Consistent with a role for Shs1 dephosphorylation in regulating septin organization at this stage of the cell cycle, when cells lacking Rts1 are propagated at the stressful temperature of 37°C, split rings are misshapen, fail to disassemble properly when a new bud emerges and, more often then not, cytokinesis is not successfully completed [53]. Interestingly, under certain experimental conditions, a single yeast cell can possess multiple buds, and the septin structure that is present at each neck is appropriate to the extent to which that bud has matured [54]. This observation provides suggestive evidence that septin assembly and dynamics are largely influenced by modifications exerted locally rather than responding solely to signals imposed globally across the cell. This same situation is certainly the case in filamentous fungi, like *Ashbya gossypii*, in which it has been shown that, despite little spatial or temporal separation between them, numerous distinctly different septin-based structures can co-exist in a shared cytoplasm and are subject to regulation by distinct kinases (including the Gin4 ortholog) [55]. Thus, reversible modifications drive transitions in higher-order septin structure, and an inappropriate state of modification (rather than persistence of any septin *per se*) is deleterious to proper coupling of morphogenesis to cell cycle progression.

### Ageism: Septins Do Not Discriminate

As described above, yeast septins are long-lived and re-used in multiple successive divisions. Thus, in each cell, molecules synthesized *de novo *("new/naive" septins) co-exist with a substantial population of pre-existing molecules ("old/experienced" septins) that have undergone at least one round of cell cycle-dependent modifications. This situation raises the possibility that old and new septins might be differentially marked, and/or spatially segregated within cellular structures, and thus unequally distributed between a mother cell and its daughter during cell division.

It is known that asymmetric segregation of certain components within other complex macromolecular assemblies can have important consequences. For example, the budding yeast centrosome equivalent, called the spindle pole body (SPB), duplicates in a conservative manner, producing an "old" and a "new" SPB [56]. The old SPB is always the one that is directed bud-ward because cytoplasmic microtubules within the mother cortex direct a regulator of spindle function (Bfa1-Bub2 GAP) specifically to the old SPB [56]. A similar mechanism regulates differential use of the SPBs in the four haploid nuclei produced during meiosis of a diploid yeast cell. As in mitosis, the first two meiotic SPBs differ slightly in age, and both are older than the SPBs generated in the second meiotic division. The temporal order in which the four SPBs are generated dictates the opportunity they have to associate with a packaging factor (Nud1/centriolin), thereby influencing the probability of when they will be encapsulated into spores [57]. Thus, in biology, molecular history can influence subsequent physiological function.

**Figure 3:**
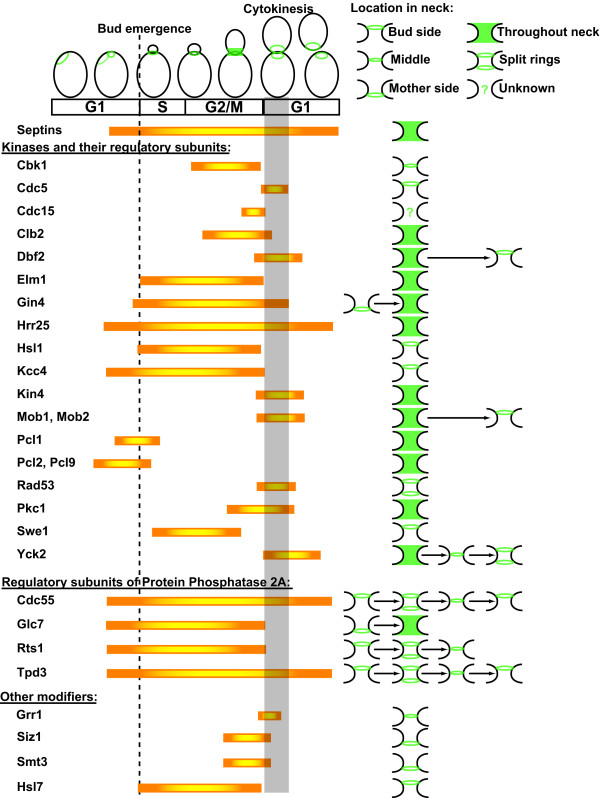
**Spatial and temporal organization of protein-modifying enzymes at the bud neck of *Saccharomyces cerevisiae***. Gene products known or predicted to have the capacity to modify other proteins and that have been visualized at the bud neck by fluorescence microscopy are listed along with the stages of the mitotic division cycle at which they are found at the neck *(orange bars*), and the region of the bud neck to which they localize *(green*), where known. Also indicated are the time when emergence of the bud first becomes visible (*dashed lined*) and the time period corresponding to disassembly of the mitotic spindle and completion of the septum (*grey bar*). Smt3 is the yeast ortholog of SUMO. It should be noted that this list does not include certain enzymes known to act on septins with important functional consequences (*e.g*, Cla4 [10]) that do not stably associate with the bud neck, and instead localize to, but quickly depart from, the future site of bud emergence [78]. See Table 1 for citations of the appropriate supporting literature. Adapted from [60] with permission from Elsevier.

As determined by fluorescence polarization measurements (similar to those described above that were undertaken to attempt to discern the orientation of the bud neck filaments *in vivo*), septin collars and rings do not appear to exhibit any internal asymmetry with respect to organization of their constituent subunits [58], in agreement with the two-fold rotational symmetry of Cdc11–Cdc12–Cdc3–Cdc10–Cdc10–Cdc3–Cdc12–Cdc11 rods and the non-polar filaments that result from their end-on-end polymerization [4]. Nevertheless, the septin-containing collar at the bud neck must be spatially asymmetric at some level, as evidenced by the fact that many of the 130 other neck-associated proteins identified to date localize either to the mother side or to the bud side of the collar [59,60] (Figure 3). Also, during a brief period after the collar has assembled, only the mother side of the collar becomes modified by covalent attachment of the C terminus of Smt3 (yeast SUMO) in isopeptide linkage to the ε-amino group of certain Lys residues in Cdc3, Cdc11 and Shs1 [61], but not Cdc10 and Cdc12. SUMOylation disappears from the bud neck just before the collar splits at the onset of cytokinesis [61] (Figure 2). However, conditions that abnormally elevate or affect the timing of septin SUMOylation have remarkably little consequence. These include preventing normal septin deSUMOylation [62], causing septin SUMOylation on both sides of the neck [62], and forcing SUMOylation of Cdc10 and Cdc12 [63]. Likewise, eliminating septin SUMOylation has no strikingly adverse effect on cell cycle progression [61,63]. Thus, despite the level of asymmetry exhibited by this modification during a normal cell division cycle, SUMOylation does not seem to play a critical structural or regulatory role in septin collar function.

Interestingly, another modification that occurs on Lys residues, and could be mutually exclusive with SUMOylation, is N-acetylation. In this regard, it is noteworthy that the protein-Lys N-acetyltransferase Eco1/Ctf7 is one component identified by mass spectrometry in protein complexes co-purifying with the septin-associated protein kinase Gin4 [49]. Similarly, it has been reported that absence of an otherwise non-essential subunit of the NuA4 histone N-acetyltransferase is synthetically lethal in cells lacking another septin-associated protein kinase, Cla4 [64]. Finally, at least in mammals, the initiator Met is removed from certain septins by the action of methionine exopeptidase and the resulting exposed α-amino group is N-acetylated [65,66]; a predictive algorithm suggests that all five mitotic *S. cerevisiae *septins may undergo the same modification [67].

Certain of the neck-associated protein kinases known to modify septins are restricted to the one side of the neck or the other, suggesting that particular phosphorylation events may also show such a separation. For example, Gin4 (1142 residues) starts off on the mother side of the collar [60] (Figures 2 and 3), whereas the closely-related (74% identity) enzyme Kcc4 (1032 residues) is found exclusively on the bud side of the collar [68] (Figure 3). Establishment of this strikingly asymmetric localization occurs early, during assembly of the ring of septins that marks the incipient bud site. For example, septin-binding protein Bni4 associates with the exterior of the ring, whereas Kcc4 is located only at the interior of the ring [68]. Theoretically, if old and new septins were differentially marked, unequal deposition of old and new septins on the outer and inner aspects of the ring (or further differential modifications at the outer and inner edges of the ring) could contribute to establishment of the distinctions between these two zones. In any event, mother-bud asymmetry in septin structures does not appear to be based on any polarity in the organization or arrangement of the constituent septins themselves, but seems instead to be a function of their modification state and/or the nature of their interaction partners.

To address whether differential use of old and new septin molecules might contribute to generating the observed asymmetries, a pulse-chase approach that permits the attachment of fluorescent labels, at will, to existing pools of septin-SNAP-Tag™ fusion proteins was used to distinguish newly synthesized from pre-existing molecules [42]. In the septin structures formed in mitotically dividing cells, new and old septins were found to be intermixed rather homogenously, at least at the resolution of light microscopy [42]. Additionally, old septins were equipartitioned between mother and daughter at each division [42]. Thus, unlike other cellular components, older septins do not accumulate in aging mother cells, even though, ironically enough, trapping other aged, worn-out and damaged cellular components in the mother cell is dependent on the diffusion barrier imposed by the septin collar at the bud neck [12]. The conclusions reached by using time-dependent labeling of SNAP-tagged septins, namely that old septin proteins are reused and recycled many times and and co-localize with newly-made septins, was corroborated using an independent approach for producing and distinguishing between old and new septin based on differential expression of GFP- and mCherry-tagged septins [42].

The observed intermixing is also consistent with analyses of septin structures performed using fluorescence recovery after photobleaching (FRAP), which indicated extensive mobility of subunits within septin structures at various stages of the cell cycle [53,69]. Importantly, however, the FRAP method cannot distinguish whether the mobile entity is an individual fluorophore-tagged septin or a larger multimeric complex that contains it. *In vitro*, purified Cdc11–Cdc12–Cdc3–Cdc10–Cdc10–Cdc3–Cdc12–Cdc11 octameric rods are quite stable and resist dissociation even in buffers of high ionic strength (*e.g*., 1 M KCl) [4,20-22], in agreement with the cumulative evidence that such rods are the fundamental building block of septin filaments and higher-order structures seen *in vivo*. Nonetheless, to examine at molecular resolution whether such rods are stable *in vivo *once formed, or whether new subunits can be exchanged for old in pre-formed rods, cells expressing a SNAP-tagged septin were pulse-labeled to completion with a biotin affinity label, allowed to assemble into rods, and then allowed to mature through several yeast cell cycles, during which time new (unlabeled) SNAP-tagged molecules are synthesized. The cells were then lysed in high salt and streptavidin capture was used to recover the rods that contain the old (biotin-labeled) septin-SNAP tag subunits. It was found that the majority of these rods also contained newly-made SNAP-tagged subunits, as judged by the fact that they could be labeled subsequently *in vitro *by incubation with a reactive dye directed against the unoccupied SNAP-tags in those new molecules [42]. Thus, this observation suggests that, in the cell, the subunits within preformed rods undergo dynamic exchange (Figure 2).

### Septin Inheritance During Meiotic Divisions

As already recounted above (Figure 2), the transitions of the yeast mitotic division cycle are accompanied by a series of discrete septin-based structures. However, the yeast life cycle includes other development options that also involve formation of unique septin-containing structures distinct from those in mitotic cells. Haploid cells of opposite mating type pair and fuse to form diploids, which can undergo meiosis and sporulation to generate haploid spores. In haploids responding to mating pheromone, normal budding and collar formation are abrograted and septins are found instead at the base and flanks of the polarized structure (mating projection) that forms in such cells. As already mentioned earlier, septin-based structures are formed at the leading edge of the developing spore membranes. Certain septins are essential for all of these events in the yeast life cycle, raising the question of whether a septin subunit made during mitotic division can be recycled for use toward a different developmental purpose, or whether those pre-made proteins are discarded and only newly-made ones employed for such developmental transitions. When the behavior of fluorescent septins, generated by pulse-labeling of septin-SNAP tag fusions, was monitored throughout the course of sporulation, three distinct fates were revealed, depending on the subunit [42]. One subunit (Cdc10) was reused and recycled – that is, molecules synthesized during mitotic proliferation were reincorporated alongside new molecules made during sporulation to build structures near the developing spore membranes. In contrast, a second subunit (Cdc12) made prior to the induction of meiosis also persisted during sporulation, but was not incorporated into the septin-containing structures around the developing spores. Instead, these old Cdc12 molecules were relegated to the ascal cytoplasm and not encapsulated into spores; thus, upon spore germination, the Cdc12 molecules that populate the septins structures needed to support mitotic proliferation were generated only by *de novo *synthesis. Finally, a third subunit (Spr3) was expressed only during sporulation and replaced the mitosis-specific subunit Cdc12 within the septin complexes in meiotic cells; upon spore germination, robust synthesis of Cdc12, and lack of any further production of Spr3, results in its replacement by Cdc12, thereby excluding Spr3 from mitotic structures. Conversely, robust production of Spr3 in meiotic cells, combined with diminished Cdc12 expression, may contribute, perhaps along with as yet unknown modifications or factors, to excluding Cdc12 from the septin structures on prospore membranes, even in the absence of its proteolytic destruction. In any event, these studies show that dynamic exchange of subunits into and out of septin complexes also occurs during developmental transitions, as well as during the mitotic cell division cycle.

### Septins and Histones: Common Principles of Assembly and Inheritance?

It is worth considering the mechanisms of septin assembly and inheritance in light of what is also known about other repeating multi-subunit structures conserved in eukaryotic cells. One such example is the nucleosome, which comprises ~165 base-pairs of double-stranded DNA wrapped around a spherical oligomer of histones, and is the fundamental building block of chromatin and higher-order chromosome structure. Just as the septin hetero-octamer in yeast comprises two copies of each of four different classes of subunits, the nucleosome core is composed of two copies of each of four different classes of histone. Just as the residues in septins can be heavily modified post-translationally, the histones are especially heavily decorated by a variety of post-translational modifications that regulate, among other things, nucleosome accessibilty, higher-order chromatin structure, and coordination of chromosome organization with progression through the cell cycle. Like the septins examined to date, the histones are extremely long-lived in most cellular circumstances [70,71], demanding that the relevant covalent modifications be reversible. Indeed, existing histone modifications can be enzymatically removed (or counteracted by additional modifications) without disrupting the nucleosome core itself [72,73], providing non-destructive ways to alter chromatin structure. As observed for septin complexes, exchange of subunits within a nucleosome would allow for replacement of particular subunits with copies carrying a different array of modifications, or with histone variants encoded by distinct genes [74]. The latter echoes the substitution of sporulation-specific septins for mitosis-specific subunits observed in yeast. As is the case with septins, mitotic nucleosome inheritance is symmetrical in the general sense, *i.e*., the daughters receive an equal share of both the pre-existing and the newly-made histones [72,73]. However, at higher resolution, the degree to which nucleosomal duplication during S phase is conservative or dispersive remains controversial. Specifically, it has not been definitively established whether the 2:2 tetrameric H3:H4 subcomplex always remains intact or can, in certain situations, split into H3:H4 dimers [75]. A similar uncertainty surrounds septin hetero-octamer dynamics. Are there subunit pairs or sub-complexes that remain associated throughout the lifetime of the constituent proteins (see Figure 2)? Future studies, especially those exploiting recent advances in covalent protein labeling technology, are needed to resolve these issues.

## Conclusion

In budding yeast, septin-based structures impose restrictions on the localization of a large number of cellular factors, thereby influencing their distribution and fate during cell division. This influence extends to factors with which the septins do not physically interact and, thus, septin filaments serve not only as scaffolds, but as diffusion barriers. Collectively, by these attributes, septin structures serve as potent cortical organizers. The supramolecular architecture of septin-containing structures themselves undergoes highly regulated transitions coordinated with the yeast cell division cycle and other stages of the life cycle of this organism. During developmental transitions, pre-existing molecules of some subunits inherited from prior cell states are recycled and incorporated into complexes that also contain newly synthesized molecules of the same subunit, whereas incorporation of certain other subunits is restricted to a particular stage and can be irreversibly blocked during developmental transitions. It appears that mechanisms uncovered for regulating septin assembly, dynamics, function and inheritance display principles germane to the behavior of other cellular structures composed of multi-component complexes capable of self-association into polymers.

## Abbreviations

(GTP): guanosine triphosphate; (PH): pleckstrin homology; (GAP): GTPase activating protein; (G1): growth phase 1; (S): synthesis phase; (G2): growth phase 2; (M): mitosis.

## Competing interests

The authors declare that they have no competing interests.

## Authors' contributions

MAM composed the original manuscript, JT made extensive revisions, and both authors read and approved the final version.
